# Epithelial response to a high-protein diet in rat colon

**DOI:** 10.1186/s12864-017-3514-z

**Published:** 2017-01-31

**Authors:** Martin Beaumont, Mireille Andriamihaja, Lucie Armand, Marta Grauso, Florence Jaffrézic, Denis Laloë, Marco Moroldo, Anne-Marie Davila, Daniel Tomé, François Blachier, Annaïg Lan

**Affiliations:** 1 UMR Physiologie de la Nutrition et du Comportement Alimentaire, AgroParisTech, INRA, Université Paris-Saclay, 16 rue Claude Bernard, 75005 Paris, France; 2 UMR1313 Génétique Animale et Biologie Intégrative, INRA, 78350 Jouy-en-Josas, France; 3 CRB GADIE, INRA, 78350 Jouy-en-Josas, France

**Keywords:** Epithelial cells, Colon, Dietary protein, High-protein diet, Transcriptome, DNA damages, Barrier function, Mucus, Epithelial renewal

## Abstract

**Background:**

High-protein diets (HPD) alter the large intestine microbiota composition in association with a metabolic shift towards protein degradation. Some amino acid-derived metabolites produced by the colon bacteria are beneficial for the mucosa while others are deleterious at high concentrations. The aim of the present work was to define the colonic epithelial response to an HPD. Transcriptome profiling was performed on colonocytes of rats fed an HPD or an isocaloric normal-protein diet (NPD) for 2 weeks.

**Results:**

The HPD downregulated the expression of genes notably implicated in pathways related to cellular metabolism, NF-κB signaling, DNA repair, glutathione metabolism and cellular adhesion in colonocytes. In contrast, the HPD upregulated the expression of genes related to cell proliferation and chemical barrier function. These changes at the mRNA level in colonocytes were not associated with detrimental effects of the HPD on DNA integrity (comet assay), epithelium renewal (quantification of proliferation and apoptosis markers by immunohistochemistry and western blot) and colonic barrier integrity (Ussing chamber experiments).

**Conclusion:**

The modifications of the luminal environment after an HPD were associated with maintenance of the colonic homeostasis that might be the result of adaptive processes in the epithelium related to the observed transcriptional regulations.

**Electronic supplementary material:**

The online version of this article (doi:10.1186/s12864-017-3514-z) contains supplementary material, which is available to authorized users.

## Background

Colonic epithelium faces a highly complex mixture of residual nutrients, resident bacteria and their metabolites. Modifications of the luminal environment are known to affect the colonic epithelium and may result in colonic homeostasis perturbation by alteration of the barrier function, modification of the epithelial renewal and impairment of the mucosal immune response [1]. Among several environmental factors, dietary changes have been shown to affect the colon luminal environment and mucosal homeostasis [2].

HPD have a significant effect on the large intestine luminal ecosystem. Indeed, HPD increase the amount of undigested peptides that reach the large intestine [3–5], modify the gut microbiota composition [6–8] and increase protein fermentation by the large intestine bacteria [8–10] resulting in the production of numerous amino acid-derived metabolites [11, 12]. Among them, ammonia, *p*-cresol and hydrogen sulfide have been found to inhibit colonocyte respiration, when present at high concentrations [10, 13, 14]. In addition, *p*-cresol has been shown to induce DNA damage in colonocytes [13]. In contrast, other amino acid-derived metabolites such as indolic compounds contribute to the maintenance of epithelial homeostasis [15, 16].

These HPD-induced changes in the colonic luminal environment have been associated with several effects on the large intestine mucosa. In rats fed an HPD, the height of colonocyte brush border (a key functional feature of absorptive cells) is markedly reduced probably in relation with the observed perturbation of mitochondrial metabolism [10]. An HPD also modifies goblet cell distribution in rat colonic epithelium together with an increased gene expression of mucin 3 (*Muc3)* [17] while it does not change colonic mucosal immune response except for a decreased interleukin-6 (*Il-6*) mRNA expression [17]. In HPD-fed piglets, mucin gene expression as well as pro- and anti-inflammatory cytokines are upregulated in the colonic mucosa without modification of the histological aspect [18, 19]. In this latter animal model, there is no change in colonic barrier function after the HPD [20]. Conflicting results, according to the model and/or the type of HPD used, have been reported regarding the induction of DNA damage in colonocytes of rats fed an HPD [21–24]. Recently, it was concluded from microarray experiments that an HPD upregulates in rat colonic mucosa the expression of genes implicated in glutathione metabolism, chemotaxis, tumor necrosis factor-α signaling and apoptosis while it downregulates genes related to oxidative phosphorylation, glycosylation of mucins and innate immune responses [8]. Since the colonic mucosa contains a cell mixture from epithelium, lamina propia and muscularis mucosae layers, it is not possible to determine from that study what are the effects of HPD specifically on colonic epithelial cells that are directly exposed to luminal changes induced by these diets.

In this context, the aim of the present study was to characterize the epithelial response to a whole milk protein-based HPD in rat colon compared to an NPD. The experimental settings were similar to our previous studies showing an HPD-induced modulation of the microbiota composition and of the luminal bacteria metabolite content in the rat large intestine [7, 10, 17]. Gene expression signature was determined by transcriptome profiling in combination with cellular and functional analysis to define the effects of an HPD consumption at the colonic epithelial level.

## Methods

### Animals and diet

The present protocol received written agreement from the local animal ethical committee (COMETHEA at Jouy-en-Josas, France, N°12/090). Male Wistar rats (Harlan, Gannat, France) weighing 150 g (5–6 weeks) were fed for 1 week a standard rodent diet containing 16% protein by weight. Subsequently, 16 rats received for 15 days either an NPD (*n* = 8) or an HPD isocaloric (*n* = 8) adjusted on digestible carbohydrates (Table 1) and water ad libitum. The animals were maintained in a 12:12-h light-dark cycle with the dark period beginning at 7:00 PM. At the end of the experiments, rats were anesthetized with pentobarbital sodium (40 mg/kg body weight). The whole colon was isolated and the last 2 cm-segment was used for Ussing chamber experiments or for histology examination. The remaining part of the colon was used for colonocyte isolation.

**Table 1:** Composition of the experimental diets

Ingredients (g/kg)	NPD^a^	HPD^a^
Whole milk proteins	140	530
Corn starch	622.7	287.0
Sucrose	100	45.7
Cellulose	50	50
Soybean oil	40	40
Choline	2.3	2.3
Vitamin mixture, AIN 93-V	10	10
Mineral mixture, AIN 93-M	35	35
Energy, kJ/g	14.6	14.6

^a^ *NPD* normal-protein diet, *HPD* high-protein diet

### Colonocyte isolation

Colonic epithelial cells (colonocytes) were isolated as previously described [25]. Briefly, colon was flushed with a NaCl 9 g/l solution and then with a Ca^2+^ and Mg^2+^ -free Krebs Henseleit bicarbonate (pH 7.4) buffer solution containing 10 mM HEPES, 5 mM DTT, and 2.5 g bovine serum albumin and equilibrated against a mixture of O_2_ and CO_2_ (19:1, vol/vol). Then, colon was perfused for 20 min at 37 °C with the same buffer containing 10 mM EDTA. Colonocytes were detached by gently tapping the colon.

### RNA extraction from isolated colonocytes and microarray experiment

After three washes in PBS by centrifugation (200 g, 3 min), isolated colonocytes were immediately homogenized in Trizol and stored at -80 °C prior total RNA extraction [26]. A subsequent step of RNA purification was performed using the RNeasy Mini Kit and DNase I (Qiagen). RNA integrity was checked using a 2100 Bioanalyzer (Agilent Technologies). Sixteen colonocyte RNA samples (8 from NPD and 8 from HPD groups) were used for microarray experiment with SurePrint G3 Rat Gene Expression v2 8x60K Microarrays (AMADID: 028279) according to the manufacturer instructions (Agilent Technologies). Differential analysis of transcriptomic data was performed with the Bioconductor R package Limma [27]. Data were normalized with a log2 transformation, centered by array and replicate spots were averaged. Principal component analysis revealed two outliers (one in each group) that were removed from further analysis (Additional file 1: Figure S1). A linear model was fitted using *lmFit* function, with the diet as a fixed effect. Empirical Bayes approach was used to compute *p*-values and change in gene expression (*eBayes* function). Correction for multiple testing was done with the Benjamini-Hochberg procedure [28]. Differentially expressed (DE) genes selected with the adjusted *p*-value (q) cut-off q < 0.1 were used for pathway analysis using Ingenuity Pathway Analysis Software (Qiagen, Build version 355958 M, Content version 24718999, Release Date 2015-09-14). A flow diagram of the transcriptome analysis is presented in Additional file 2: Figure S2. The data have been deposited in the Gene Expression Omnibus with the accession number GSE83953.

### Real-time PCR

Quantitative real-time polymerase chain reaction (qRT-PCR) was performed to validate microarray experiment using a set of rat-specific primers (Eurogentec) which were designed using the Oligo Explorer 1.1.0 software (GeneLink), based on published sequences of the target genes (sequences available on request). After cDNA synthesis from mRNA using the High Capacity cDNA Reverse Transcription Kit (Life Technologies), qRT-PCR was performed using the power SYBR Green PCR master mix on a StepOne Real-Time PCR system platform (Life Technologies). Gene expression levels for each sample were normalized relative to the *Hprt* gene, using the 2^−ΔΔCt^ calculation.

### Comet assay

DNA strand breaks and alkali-labile sites in isolated colonocytes were assessed using the alkaline version of the comet assay. Cells were rinsed with PBS and pelleted by centrifugation for 3 min at 200 g three times before re-suspension in sucrose 85.5 g/L, DMSO 50 mL/L prepared in citrate buffer (11.8 g/L), pH 7.6, and immediately frozen at -80 °C. Three microscope slides per condition were coated with 1% normal melting point agarose (NMA) and allowed to dry. Ten thousand cells per slide were mixed with 0.6% low melting point agarose (LMPA) and deposited over the NMA layer. The cell/LMPA mix was then allowed to solidify on ice for 20 min. Slides were immersed in lysis solution (2.5 M NaCl, 100 mM EDTA, 10 mM Tris, 10% DMSO, 1% Triton X-100) overnight at 4 °C, before being rinsed in 0.4 M Tris pH 7.4. DNA was then allowed to unwind for 1 h in alkaline electrophoresis solution (300 mM NaOH, 1 mM EDTA, pH > 13). Electrophoresis was performed in an electric field of 21 V and 300 mA for 20 min. Slides were then neutralized in 0.4 M Tris pH 7.4 and were stained with 20 μL of 10 000 X diluted Sybr Gold (Life Technologies). Fifty μM H_2_O_2_ (positive control) were directly deposited onto the agarose layer containing the cells and were incubated for 20 min at 37 °C. At least 50 comets per slide were analyzed under a fluorescence microscope (Carl Zeiss) connected to a charge-coupled device camera with a 350–390 nm excitation and 456 nm emission filter at x 20 magnification. Comets were measured and analyzed using Comet IV software (Perceptive Instruments).

### Histology

After an overnight fixation, 0.5 cm sections of distal colon were embedded in paraffin wax. Immunohistochemistry Ki67 labelling was carried out on 4 μm-transversal colon sections at the Cochin HistIM Facility. After antigen unmasking in sodium citrate buffer 10 mM pH 6.0, expression of Ki67 was detected using an anti-Ki67 antibody (ab15580, Abcam, dilution 1:500) and counterstained with hematoxylin and eosin.

### Western blot

Isolated rat colonocytes were lysed in RIPA buffer containing a protease inhibitors cocktail (Roche). Total protein extracts (30 μg) were loaded into 4–12% Criterion XT gel (Bio-Rad) before electrophoresis in MOPS buffer (Bio-Rad). After transfer on nitrocellulose membrane and incubation in blocking solution (TBS pH 7.5, 0.05% Tween 20 and 5% (weight:volume) BSA, membranes were incubated overnight (4 °C) with a primary antibody directed against activated-caspase 3 (Abcam 2303, rabbit, 1/1000) or proliferating cell nuclear antigen (PCNA, Abcam 29, mouse, 1/1000) or claudin-1 (Invitrogen, 717800, rabbit, 1/250) diluted in the blocking solution. After washes, blots were incubated for 2 h at room temperature with an anti-rabbit or anti-mouse HRP-linked secondary antibody (Jackson Immuno Research Laboratories, 1/5000) or a goat anti-actin-HRP (Santacruz Biotechnologies C-11, 1/1000) diluted in the blocking solution. After 3 washes, detection was performed by chemiluminescence using Clarity Western ECL substrate (Biorad) and the FluorChemFC2 device with AlphaView software (Cell Biosciences).

### Ussing chambers experiments

Rat distal colon was mounted in the EasyMount (Physiologic Instrument Inc.) Ussing chambers with an exposed area of 0.5 cm^2^. Tissues were bathed in Krebs-Ringer bicarbonate buffer (KRB) with the following composition (in mM): 120 NaCl, 4.6 KCl, 0.5 MgCl_2_, 0.7 Na_2_HPO_4_, 1.9 NaH_2_PO_4_, 15 NaHCO_3_ and 1.2 CaCl_2_. Serosal KRB contained 10 mM glucose (pH 7.35) and mucosal KRB, 10 mM mannitol (pH 7). Each chamber side was gassed with 95% O_2_ – 5%CO_2_ and kept at 37 °C. After 15 min equilibration, the trans-mural potential (*V*_*t*_, mV) was continuously recorded in open circuit configuration by the automated dual-channel epithelial voltage clamp EC825A (Warner Instruments LLC). Each 15 min, the tissues were successively voltage-clamped to zero to obtain the short-circuit current (*I*_sc_, μA/cm^2^) and a ± 5 μA current was pulsed to measure the trans-mural electrical resistance (*R*_*t*_, ohm∙cm^2^). Analogue signals were digitized using the PowerLab® 8/35 data acquisition system and analyzed with the LabChart® software (AD Instruments).

FITC dextran (FD4, Sigma) was used to evaluate the epithelial barrier integrity. After the 15 min tissue equilibration step, FD4 was added to the chamber mucosal side at the final concentration of 0.2 mM.100 μL aliquots were collected from the serosal side every 15 min over two h and replaced with 100 μL of fresh KRB. Fluorescence was measured with the Infinite® 200 Pro spectrofluorimeter (Tecan) with an excitation and emission wavelengths of 490 nm and 520 nm respectively, and FD4 amounts were calculated against a FD4 standard curve. The FD4 apparent permeability coefficient (*P*_*app*_, cm/s) was determined using the following equation: *P*_*app*_ = (*dQ/dt*) x (1/*AC*_*0*_) where *dQ/dt* is the FD4 transport rate across the epithelium (mmol/s), *A* is the exposed surface area (cm^2^), *C*_*0*_ is the initial FD4 concentration in the mucosal compartment (mmol/mL).

### Statistical analysis

Statistical analyses were performed with Prism 7 sofware (GraphPad). Mean values of measured parameters in NPD and HPD-fed rat groups were compared with an unpaired *t*-test. Differences with *p*-values < 0.05 were considered as statistically significant.

## Results

**Fig. 1:**
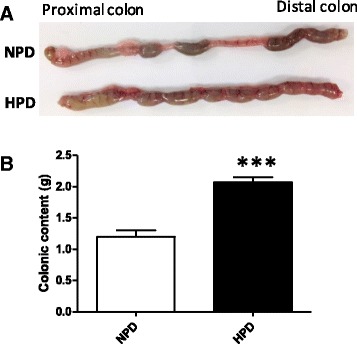
**a** Macroscopic aspects of colon from rats fed a normal-protein diet (NPD) or a high-protein diet (HPD). **b** Colonic content weight. **a**-**b** Data presented on histograms are means +/- S.E.M. Mean values were compared with a *t* test. ***: *p* < 0.001

As shown in Fig. 1a, the macroscopic aspect of colon from rats fed an HPD or an NPD were markedly different. Colons from rats fed an HPD were distended due to a very large increase in luminal content (Fig. 1b). This observation supports the hypothesis that the HPD deeply reshaped the luminal environment in contact with epithelial cells.

### Effects of the high-protein diet on transcriptomic profile in colonocytes

Microarrays were used to compare the transcriptome profile in colonocytes from rats fed an HPD or an NPD. The analysis identified 1910 differentially expressed (DE) genes between the HPD and the NPD groups at a q-value of 0.1. Among these genes, 646 were upregulated by the HPD while around twice more genes (1264) were downregulated by the HPD. Fold changes in DE genes between HPD and NPD-fed rats ranged from 5.85 (*Mt2a*, metallothionein 2A) to -2.09 (*Slc39a4*, solute carrier family 39 zinc transporter, member 4). The 20 top DE genes between HPD and NPD-fed rats are listed in Table 2 while the full list is shown in Additional file 3: Table S1. In total, 315 genes were upregulated with a fold change > 1.2 while 1103 were downregulated with a fold change < -1.2, and 492 had limited changes in expression level (-1.2 < fold change < 1.2). To validate microarray data, the expression level of four genes of interest was measured by qRT-PCR (Additional file 4: Figure S3). The results confirmed that *Tfrc* (transferrin receptor) and *Mt1a* were upregulated in the HPD group while *Slc39a4* and *Sdc4* (syndecan 4) were downregulated (q < 0.1).

**Table 2:** Most differentially expressed genes in colonocytes isolated from rats fed a high-protein diet compared to rats fed a normal-protein diet

Gene Symbol	Gene name	Fold change	q-value
*Slc39a*	Solute Carrier Family 39 (Zinc Transporter), Member 4	- 2.09	0.001
*Cela1*	Chymotrypsin-Like Elastase Family, Member 1	-1.97	0.049
*Ppp2r2a*	Protein Phosphatase 2, Regulatory Subunit B, Alpha	-1.97	0.012
*Cpd*	Carboxylpeptidase D	-1.92	0.034
*Hla-a*	Human Leucocyte Antigen-A	-1.80	0.013
*Ostc*	Oligosaccharyltransferase complex subunit (non-catalytic)	-1.76	0.035
*Ndrg1*	N-myc downstream regulated 1	-1.75	0.069
*Pnrc2*	Proline-rich nuclear receptor coactivator 2	-1.73	0.043
*Prdx1*	Peroxiredoxin 1	-1.72	0.078
*Rpl5*	Riposomal protein L5	-1.71	0.052
*Pdha2*	Pyruvate dehydrogenase E1 alpha 2	1.63	0.052
*Prap1*	Proline-rich acidic protein 1	1.66	0.078
*Tfrc*	Transferrin receptor	1.67	0.043
*Rab44*	Member RAS oncogene family	1.67	0.069
*Krtap13-2*	Keratin associated protein 13-2	1.67	0.035
*Znf780b*	Zinc finger protein 780B	1.68	0.013
*Mt4*	Metallothionein 4	1.84	0.034
*Mt1*	Metallothionein 1	2.90	0.012
*Spink4*	Serine peptidase inhibitor, Kazal type 4	3.06	0.049
*Mt1m/Mt2A*	Metallothionein 2A	5.85	0.001

**Fig. 2:**
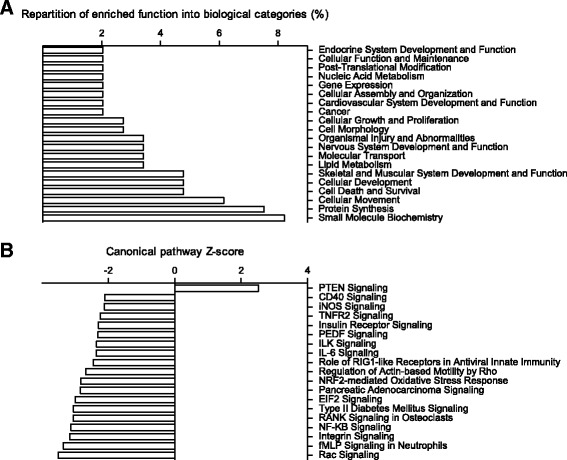
Functional analysis of differentially expressed genes in colonocytes isolated from rats fed a high-protein diet (HPD) compared to a normal-protein diet was performed with Ingenuity Pathway Analysis software. **a** Distribution of significantly enriched functions (*p* < 0.05) into biological categories is presented as a percentage of the total number of enriched functions (77). **b** Canonical pathways significantly enriched (*p* < 0.05). A pathway was considered downregulated in the colonocytes of HPD-fed rats when Z-score was < -2 and upregulated when Z-score was > 2. Canonical pathways significantly enriched but not associated with a significant Z-score are not shown

Functional analysis was performed to identify the biological pathways regulated at the mRNA level by the HPD in rat colonocytes. Seventy-seven biological functions were found to be significantly enriched in the set of genes DE between NPD- and HPD-fed rats (*p* < 0.05) (Additional file 5: Table S2). Repartition of these functions into biological categories is shown in Fig. 2a. Eighteen canonical pathways significantly enriched (*p* < 0.05) were predicted to be downregulated in colonocyte of HPD-fed rats (Z-score < -2) and only 1 was predicted to be upregulated (Z-score > 2) (Fig. 2b). The full list of significantly enriched canonical pathways is shown in Additional file 6: Table S3. Functional analysis also identified two predicted transcription regulators: MYC and E2F1, that are known to regulate, respectively, the expression of 29 and 13 genes DE between HPD and NPD-fed rats (*p* < 0.05).

### Effects of the high-protein diet on the expression of genes related to metabolism in colonocytes

Many significantly enriched functions in the set of DE genes belonged to cell metabolism-related categories. Indeed, *Small Molecule Biochemistry*, *Protein Synthesis* and *Lipid Metabolism* were among the most represented biological categories (Fig. 2a). A full list of significantly enriched functions related to cell metabolism is presented in Table 3. The majority of the genes implicated in these pathways were downregulated in colonocytes of rats fed with an HPD. Interestingly, *Protein Synthesis* was the most affected metabolic pathway according to the number of enriched functions and to the number of genes implicated in each function.

**Table 3:** Significantly enriched functions related to cellular metabolism in the set of genes differentially expressed in colonocytes isolated from rats fed a high-protein diet compared to rats fed a normal-protein diet. ‘Genes’ indicates the number of genes differently expressed implicated in the function, ‘Up’ and ‘Down’ indicate the number of genes upregulated and downregulated, respectively

*Biological category* Function annotation	*p*-value	Genes	Up	Down
*Protein synthesis*				
Polymerization of protein	1.08E-04	36	7	31
Metabolism of protein	3.52E-02	31	2	29
Oligomerization of protein	6.50E-05	30	6	24
Hetero-oligomerization of protein	1.69E-03	17	2	15
Homo-oligomerization of protein	2.12E-03	14	4	10
Hydrolysis of protein fragment	4.32E-02	12	2	9
Metabolism of peptide	1.39E-02	10	0	10
Translation	6.87E-03	9	0	9
Assembly of protein-protein complex	5.90E-03	8	0	8
Translation of protein	1.83E-02	8	0	8
Folding of protein	1.29E-03	7	1	6
Translation of mRNA	1.71E-02	7	0	7
Metabolism of neutral amino acid	3.38E-02	3	0	3
Transport of D-serine	9.13E-03	2	1	1
*Lipid metabolism*				
Synthesis of glycolipid	3.88E-02	6	1	5
Beta-oxidation of fatty acid	4.56E-02	6	1	5
Synthesis of long chain fatty acid	4.81E-02	2	0	2
Uptake of long chain fatty acid	4.81E-02	2	0	2
*Nucleic acid metabolism*				
Metabolism of purine nucleotide	3.31E-02	8	0	8
Metabolism of nucleoside triphosphate	3.88E-02	6	0	6
Transport of nucleoside	7.52E-03	3	0	3
*Metabolism of carbohydrate*	3.81E-02	28	3	25

### Effects of the high-protein diet on glutathione metabolism, oxidative stress and DNA damages in colonocytes

**Fig. 3:**
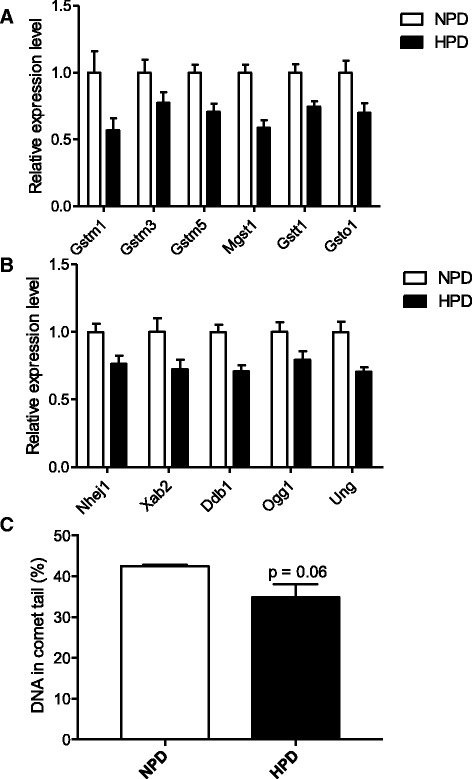
**a** and **b** Relative expression values of a selection of significantly differentially expressed genes (q < 0.01) participating to the enriched pathways *Glutathione-Mediated Detoxification* (**a**) and *DNA repair* (**b**) in the colonocytes of rats fed a high-protein diet (HPD) compared to a normal-protein diet (NPD)*.* The expression values were obtained by microarray experiment and normalized to the mean value in the NPD group. *Gstm1, 3* and *5* (glutathione S-transferase mu 1, 3 and 5), *Mgst1* (microsomal glutathione S-transferase 1), *Gstt1* (glutathione S-transferase theta 1), *Gsto1* (glutathione S-transferase omega 1), *Nhej1* (non-homologous end-joining factor 1), *Xab2* (XPA Binding Protein 2), *Ddb1* (damage-specific DNA binding protein 1), *Ogg1* (8-oxoguanine DNA glycosylase), *Ung* (uracil DNA glycosylase). **c** - DNA damages in colonocytes of rats fed an HPD or an NPD were assessed with the comet assay. The percentage of DNA in the tail of the comet is proportional to the amount of DNA damages in the cells. Mean values were compared with a *t* test. **a**-**c** Data presented are means +/- S.E.M

Two canonical pathways related to glutathione metabolism were found to be significantly enriched in the set of genes regulated by the HPD (Additional file 6: Table S3), namely *Glutathione Redox Reactions II* and *Glutathione-Mediated Detoxification*. The 8 DE genes participating to these pathways were all downregulated by the HPD as shown in Fig. 3a with selected DE genes as examples (q < 0.01). The canonical pathway of *Nuclear factor (erythroid-derived 2)-like 2 (NRF2)-Mediated Oxidative Stress Response* was also predicted to be downregulated after the HPD (Z-score: -2.83, Fig. 2b). The functional analysis revealed that the function *Repair of DNA* was significantly enriched in the set of genes DE between NPD and HPD-fed rats (Additional file 5: Table S2). Indeed, the HPD significantly decreased the gene expression of several DNA repair proteins in colonocytes (q < 0.01) (Fig. 3b). DNA damages in colonocytes were then evaluated by the comet assay. As shown in Fig. 3c, a trend towards lower DNA damage in colonocytes isolated from HPD-fed rats was observed when compared to NPD-fed rats (*p* = 0.06).

### Effects of the high-protein diet on NF-κB related pathways in colonocytes

**Fig. 4:**
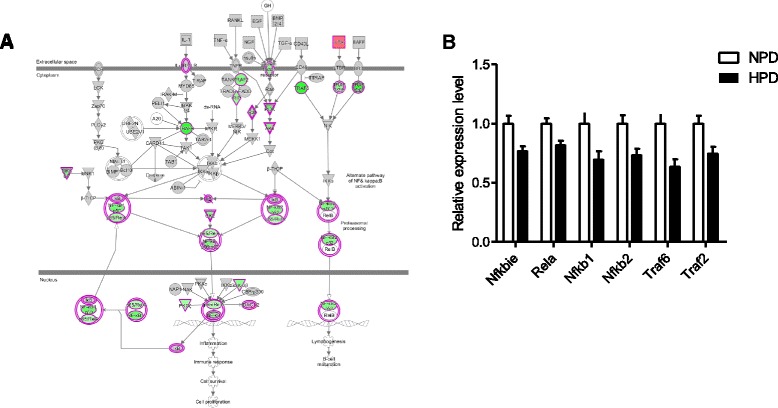
**a** *NF-κB signaling* canonical pathway diagram. This pathway was significantly enriched in the set of genes regulated by the high-protein diet (HPD). The diagram was obtained from Ingenuity Pathway Analysis software and depicts genes implicated in this pathway and their interactions. Expression of genes colored in green and red were respectively downregulated (q < 0.1) and upregulated (q < 0.1) in colonocytes of rats fed an HPD when compared to a normal-protein diet (NPD). The list of differentially expressed genes implicated in this pathway is presented in Additional file 6: Table S3. **b** Relative expression values of a selection of significantly differentially expressed genes (q < 0.01) participating to the *NF-κB signaling* pathway in the colonocytes of rats fed an HPD when compared to an NPD. The expression values were obtained by microarray experiment and normalized to the mean value in the NPD group. *Nfkbie* (NF-κB inhibitor epsilon), *Rela* (RELA proto-oncogene, NF-κB subunit), *Nfkb1* and *2* (NF-κB subunit 1 and 2), *Traf2* and *6* (TNF receptor associated factor 2 and 6). Data presented on histograms are means +/- S.E.M

An important finding of the present study was the predicted downregulation of *NF-κB Signaling* canonical pathway in colonocytes of HPD-fed rats (Z-score: -3.13, Fig. 2b). Twenty-three genes DE in colonocytes of rats fed an HPD were implicated in this pathway, among which 19 were downregulated and only 4 were upregulated (q < 0.01) (Fig. 4a and Additional file 6: Table S3). The relative expressions of several of these DE genes are shown in Fig. 4b (q < 0.01). Other NF-κB-related canonical pathways such as *IL-6* and *iNOS Signaling* were also predicted to be inhibited in colonocytes of rats fed an HPD (Z-score: -2.36 and -2.12, respectively, Fig. 2b).

### Effects of the high-protein diet on epithelium renewal in colon

**Fig. 5:**
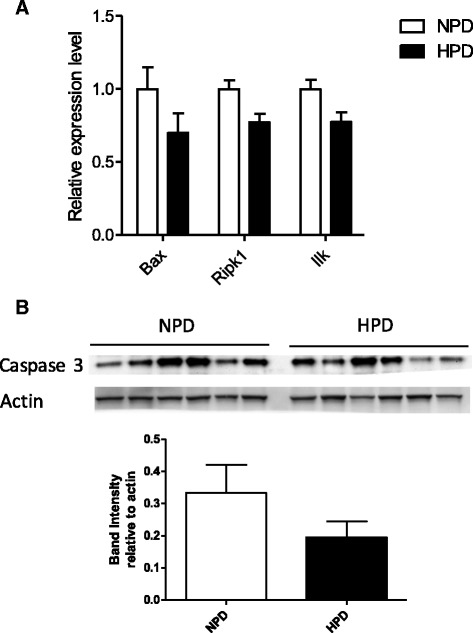
**a** Relative expression values of a selection of significantly differentially expressed genes (q < 0.01) participating to the enriched pathway *Cell death* in the colonocytes of rats fed a high-protein diet (HPD) when compared to a normal-protein diet (NPD)*.* The expression values were obtained by microarray experiment and normalized to the mean value in the NPD group. *Bax* (BCL2 associated X, apoptosis regulator), *Ripk1* (receptor interacting serine/threonine kinase 1), *Ilk* (integrin linked kinase). **b** The expression of the apoptotic marker activated caspase 3 protein was quantified by western blot in colonocytes of rats fed an HPD or an NPD. Band intensity was quantified and normalized to the intensity of the band corresponding to actin. For each protein, mean values were compared with a *t* test. **a**-**b** Data presented on histograms are means +/- S.E.M

**Fig. 6:**
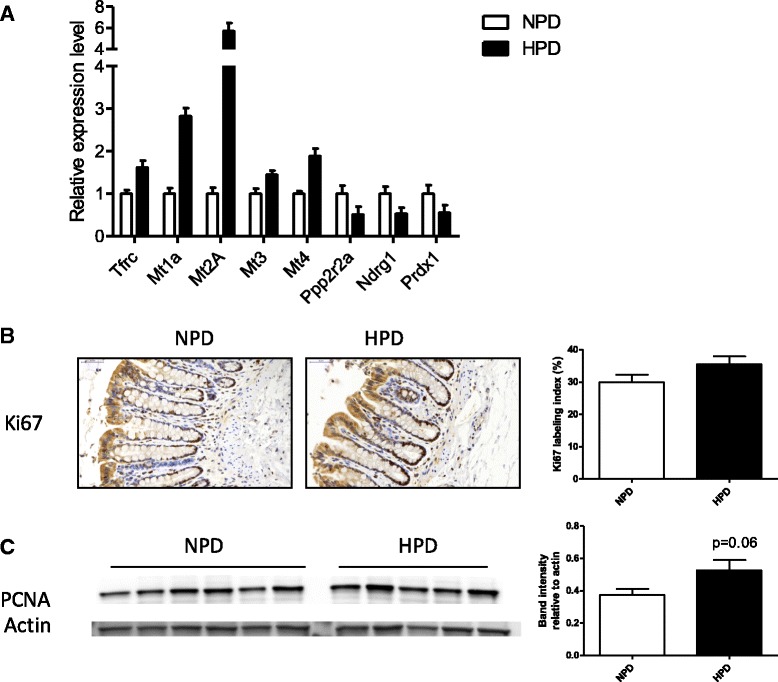
**a** Relative expression values of a selection of significantly differentially expressed genes (q < 0.01) related to proliferation in the colonocytes of rats fed a high-protein diet (HPD) when compared to a normal-protein diet (NPD)*.* The expression values were obtained by microarray experiment and normalized to the mean value in the NPD group. *Tfrc* (transferrin receptor), *Mt1a*, *2a*, *3* and *4* (metallothionein 1A, 2A, 3 and 4), *Ppp2r2a* (protein phosphatase 2 regulatory subunit B alpha), *Ndrg1* (N-myc downstream regulated 1), *Prdx1* (peroxiredoxin 1). **b** Staining of Ki67 by immunohistochemistry on distal colon of rats fed an NPD or an HPD. Ki67 labelling index was calculated as the percentage of Ki67 positive cells relative to the total number of cells within the same crypts. **c** Proliferating cell nuclear antigen (PCNA) protein expression was quantified by western blot in colonocytes of rats fed an HPD or an NPD. Band intensity was quantified and normalized to the intensity of the band corresponding to actin. **b**-**c** Mean values were compared with a *t* test. **a**-**c** Data presented on histograms are means +/- S.E.M

HPD regulated numerous functions implicated in biological processes related to *Cell Death and Survival*, *Development*, *Morphology*, *Growth* and *Proliferation* (Fig. 2a). A list of selected significantly enriched functions related to these biological categories is presented in Table 4. For all these functions, a majority of genes were downregulated by the HPD and some of them were highly relevant to epithelial renewal (*Cell Death*, *Anoïkis*, *Neoplasia of Epithelial Tissue*) (Table 4). Strikingly, the function with the greatest number of genes regulated by the HPD was *Cell Death* (103 genes, 82.5% of which were downregulated) (Table 4 and Additional file 5: Table S2). The relative expression of selected DE genes included in the function *Cell Death* are presented in Fig. 5a (q < 0.01). However, the expression of the apoptosis marker activated caspase 3 measured by western blot in colonocytes was highly variable among rats and there was no significant effect of the diet (Fig. 5b). Numerous genes known to be highly expressed in proliferating cells (*Tfrc*, metallothioneins genes) were upregulated by the HPD while tumor suppressor genes (*Ppp2r2a, Ndrg1* and *Prdx1)* were downregulated by the HPD (q < 0.01) (Fig. 6a) [29–33]. Proliferation in colonocytes was evaluated by immunodetection of Ki67 in distal colonic mucosa and by quantification of PCNA in colonocytes by western blot (Fig. 6b and c). Although visually Ki67-labelling seemed generally more intense in the crypts of rats fed an HPD, there was no significant difference with NPD-fed rats (Fig. 6b). PCNA also tended to be more expressed in colonocytes of rats fed an HPD (*p* = 0.06) (Fig. 6c).

**Table 4:** Significantly enriched functions in the set of genes differentially expressed in colonocytes isolated from rats fed a high-protein diet compared to rats fed a normal-protein diet. ‘Genes’ indicates the number of genes differently expressed implicated in the function, ‘Up’ and ‘Down’ indicate the number of genes upregulated and downregulated, respectively

Function annotation	*p*-value	Genes	Up	Down
Cell death	4.89E-02	103	18	85
Autophagy	2.96E-03	10	1	9
Neoplasia of epithelial tissue	1.83E-02	8	3	5
Senescence of cells	2.27E-02	3	0	3
Organization of mitochondria	2.27E-02	3	0	3
Anoïkis of carcinoma cell lines	9.13E-03	2	0	2
Morphology of intestinal cell lines	9.13E-03	2	0	2
Polarity of cells	4.81E-02	2	0	2

### Effects of the high-protein diet on barrier function in colon

**Fig. 7:**
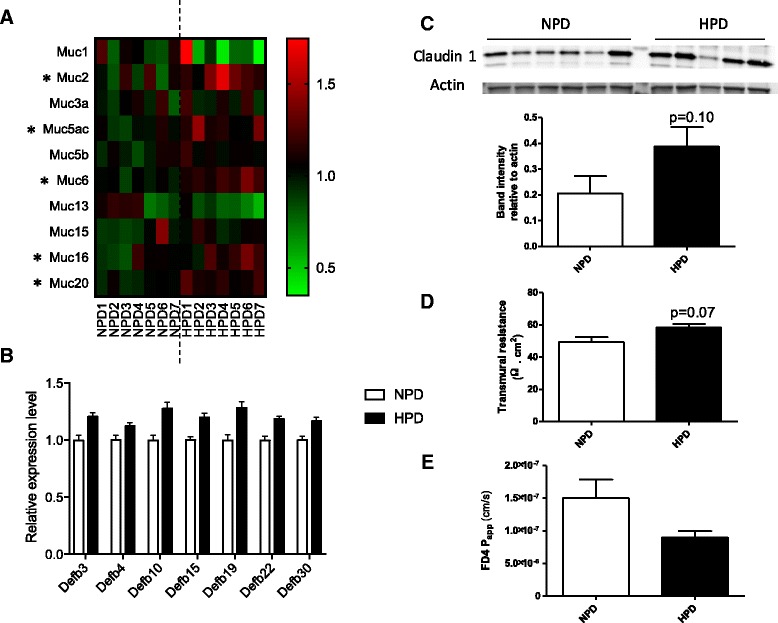
**a** Heatmap representing the expression values of mucin genes in colonocytes (*Muc*). Each row corresponds to one *Muc* gene. Each column corresponds to a single rat fed a normal-protein diet (NPD), or a high-protein diet (HPD). The color indicates the relative expression value (as indicated by the key) obtained from microarray experiment and normalized to the mean value in the NPD group. Mean expression values of the HPD and the NPD groups were compared with a *t* test. *: *p* < 0.05. **b** Relative expression values of defensin genes significantly differentially expressed (q < 0.01) in the colonocytes of rats fed a HPD when compared to a NPD. The expression values were obtained by microarray experiment and normalized to the mean value in the NPD group. *Defb3, 4, 10, 15, 19, 22* and *30* (β-defensin 3, 4, 10, 15, 19, 22 and 30). **c** Claudin 1 protein expression was assessed by western blot in colonocytes of rats fed an HPD or an NPD. Band intensity was quantified and normalized to the intensity of the band corresponding to actin. **e**-**f** Barrier function was evaluated with Ussing-chambers in distal colon of rats fed an NPD or an HDP. **e** Transmural resistance was measured for 15 min after mucosa mounting in the chamber. **f** FITC-dextran (FD4) transport from mucosal to serosal side was recorded during two hours and FD4 apparent permeability (FD4 P_app_) was calculated. **c**-**e** For each parameter, mean values were compared with a *t* test. **b**-**e** Data presented on histograms are means +/- S.E.M

Based on the predefined hypothesis that HPD could regulate mucus secretion [17, 18, 34], mucins gene expression was specifically compared in the colonocytes of rats fed an HPD or an NPD (Fig. 7a). The HPD significantly upregulated the expression of *Muc2, Muc5ac, Muc6, Muc16* and *Muc20* (*p* < 0.05). Moreover, in the list of DE genes (Additional file 1: Table S1), seven β-defensin genes were upregulated in colonocytes of rats fed an HPD when compared to an NPD (q < 0.1) (Fig. 7b). Several canonical pathways related to cell adhesion were significantly enriched in the set of genes regulated by the HPD (Additional file 6: Table S3). The *Integrin Signaling* canonical pathway was predicted to be inhibited in colonocytes of HPD-fed rats (Z-score: -3.16, Fig. 2b and Additional file 7: Figure S4). The HPD also regulated the expression of genes implicated in the following canonical pathways: *Tight Junction Signaling* (Additional file 8: Figure S5), *Actin-based Motility by Rho Family GTPases*, *ILK (Integrin Linked Kinase) Signaling*, *FAK (Focal Adhesion Kinase) Signaling* (Additional file 6: Table S3), reinforcing the possibility of an HPD-induced modification of colonocyte adhesion and interaction with extracellular matrix. However, the expression in colonocytes of the tight junction protein Claudin-1 was not significantly regulated (*p* = 0.10) by the HPD as measured by western blot (Fig. 7c). To evaluate the effect of the level of protein intake on colonic barrier integrity, distal colon segments were mounted into Ussing chambers. After 15 min, distal colon transmural resistance tended (*p* = 0.07) to be higher in rats fed an HPD when compared to rats fed an NPD (Fig. 7d) while it was similar between both groups after 60 min (data not shown). Moreover, FD4-apparent paracellular permeability across the colon wall was similar in NPD- and HPD-fed rats (Fig. 7e). In conclusion, these experiments showed that the HPD did not impair colonic barrier function in rat distal colon.

## Discussion

The results of the present study indicate that in the rat model, there is a marked colonic epithelial response to an HPD that is characterized by a specific transcriptional signature. The HPD downregulated, at the gene expression level, biological pathways related to cellular metabolism, glutathione metabolism, DNA repair, NF-κB signaling, apoptosis and epithelial cell adhesion. In contrast, genes related to proliferation and chemical barrier function were upregulated by the HPD. These transcriptional changes induced by a short-term HPD were not associated with detrimental effects on the colonic epithelium in terms of genotoxicity, epithelial renewal and barrier integrity.

Numerous genes downregulated in colonocytes by the HPD participate in cell metabolism and notably in protein synthesis. Interestingly, several bacterial metabolites derived from amino acids (*p*-cresol, hydrogen sulfide and ammonia) inhibit mitochondrial respiration when present in excess [10, 13, 14]. In addition, HPD increased proton leaks in rat colonocyte mitochondria leading to a lower energetic efficiency [10]. Therefore, it is tempting to propose that the downregulation of genes implicated in cell metabolism may represent an adaptation allowing ATP saving in colonocytes during HPD. Indeed, the colonic epithelium has a high energy need related to its constant renewal, water and electrolyte transports and barrier function maintenance [35].

Several genes related to glutathione metabolism were downregulated in rat colonocytes after the HPD. Notably, the HPD induced a decrease in gene expression of several glutathione-S-transferases (GST) which are implicated in phase II metabolism of xenobiotics but also in the control of reactive oxygen species [36]. In agreement with the present study, GST gene expression appeared to be reduced in the colon of pigs fed an HPD [18]. The HPD also induced a downregulation of genes encoding proteins involved in several DNA-repair pathways, namely base excision repair (*Ogg1*, *Ung*), non-homologous end joining (*Nhej1*) and nucleotide excision repair (*Ddb1*) [37] but this was not associated with significant changes in DNA damages in colonocytes of rats fed an HPD when compared to rats fed an NPD. However, the HPD induced a trend toward lower DNA damage in colonocytes. This result is in agreement with a recent report showing lower DNA damages in rats fed with a moderate protein intake (20%) compared to an NPD (14%) [24]. Moreover, two human studies found no increase in fecal water genotoxicity after a HPD [21, 22]. In contrast, Toden *et al* found that a casein-based or soy protein-based HPD induce DNA damages in rat colonocytes while this effect was not found with whey protein-based HPD [23]. In the present study, the HPD contained whole milk protein. These differences in protein sources used in the studies might contribute to the divergent effects of HPD consumption. Indeed, all the protein sources do not have the same digestibility, potentially modulating the quantity of dietary nitrogen entering in the large intestine [38]. However, all milk protein have very high digestibility in rats (around 96%) [39], precluding a major role of this parameter to explain the divergent reported effects of milk protein-based HPD on DNA damage. Alternatively, differences between protein sources in amino acid composition, bioactive peptides or associated microconstituents might be implicated [40].

The proliferation markers Ki67 and PCNA tended to be both more expressed in colonocytes of rats fed an HPD. This was concordant with the transcriptional overexpression in colonocytes of markers of epithelial proliferation such as metallothioneins (up to 5.84 fold change) and *Tfrc* [29, 31] while tumor suppressor genes such as *Ppp2r2a, Ndrg1* and *Prdx1* were downregulated by the HPD [30, 32, 33]. In rats fed with the HPD, the expression of the apoptosis marker caspase 3 was unchanged in colonocytes, while there was a massive downregulation of cell death-related pathways at the mRNA level. Similar results were obtained in the colon of pigs fed an HPD: PCNA being upregulated at the gene expression level while caspase 3 remaining unchanged [18]. Collectively, these data suggest that HPD act on epithelium renewal by favoring colonocyte proliferation but not apoptosis.

The HPD-induced downregulation of pathways implicated in epithelial cell junction and adhesion of colonocytes to extracellular matrix might jeopardize barrier function. However, in Ussing chamber experiments, transmural resistance and apparent permeability to FD4 (that cross the epithelium by the paracellular route) were similar in rat fed an NPD or an HPD, indicating that colonic barrier integrity was not altered. These results are consistent with our previous electronic microscopy observation showing that there was no alteration of tight junction structure in colonic epithelium of rats fed an HPD [10] and with another study showing that colonic barrier function is preserved in HPD-fed piglets [20].

A very striking result of the present study was the inhibition of the NF-κB pathway in colonocytes of rats fed an HPD. In epithelial cells, NF-κB plays a central role in immune homeostasis, epithelial renewal and maintenance of barrier function [41]. Since one of the NF-κB activation pathways is the stimulation of pattern recognition receptors by luminal bacteria, it can be hypothesized that the HPD decreased the contact between commensals and the epithelium. A lower stimulation by toxic compounds from the luminal side could also explain the decreased expression of genes related to glutathione metabolism and DNA repair observed in colonocytes of rats fed an HPD. In the present study, potential mechanisms for a reduced contact of the microbiota and its metabolites with the epithelium are the reinforcement of the mucus barrier and the increase in antimicrobial peptides secretion [42, 43]. Indeed, five mucin genes, including *Muc2* (the major gel-forming mucin), were upregulated in epithelial cells of HPD-fed rats in agreement with previous studies in rats and pigs [17, 18]. Moreover, seven β-defensin genes were overexpressed after the HPD. Since Muc2 has been shown to induce the expression of β-defensin 2 [44], the simultaneous mucin and β-defensin gene upregulations in colonocytes after the HPD might be two coordinated components of a protective adaptive response to the modifications of the luminal environment induced by the HPD [7, 10].

The results obtained in the present study are not in agreement with the transcriptome profile recently described in the colonic mucosa of rats fed an HPD [8]. Indeed, Mu et al. found that genes related to glutathione metabolism and apoptosis were upregulated by the HPD while the data of the present study indicated the opposite. For example, *Mgst1* (microsomal glutathione S-transferase 1) and *Ripk1* (receptor interacting serine/threonine kinase 1) were significantly regulated by the HPD in the two studies but with opposite direction. Three differences in the experimental designs of the two studies may explain these discrepancies. First, the present study used whole milk protein while Mu et al. used a protein isolate of casein [8]. As discussed above, the protein source might modulate the effects of HPD on gene expression in the colon. Secondly, gene expression was analyzed in the present study in isolated colonic epithelial cells while Mu et al. described the transcriptome in the whole colonic mucosa [8]. Since the gene expression patterns in the colonic epithelium and in the stroma are clearly distinct [45], the discrepancies between the two studies might be related to the different type of cells analyzed. Thirdly, the different durations of the studies (2 *versus* 6 weeks) probably also explain part of the differences in the regulation of gene expression by the HPD. Indeed, time-course microarray experiments revealed early and late transcriptomic response to dietary challenge in mice [46]. In the present study, the HPD lasted 2 weeks since previous results obtained using the same experimental model indicated that changes in gut microbiota composition and luminal bacteria metabolite content occurred within this duration [7, 10, 47].

## Conclusions

In conclusion, a 2-week HPD in rat did not impair the colonic epithelium in term of DNA-damages, epithelial renewal and barrier function. However, the transcriptional signature in colonocytes of rats fed an HPD indicates a downregulation of pathways implicated in crucial cellular processes such as NF-κB signaling, DNA repair and glutathione metabolism. These changes might be detrimental for the epithelium since a decreased expression and activity of GST is associated with colorectal cancer through a defect in carcinogen detoxification [48], unrepaired DNA damages can lead to genomic instability [37] and maintenance of an appropriate activation of NF-κB is crucial for epithelial homeostasis [41]. Further experiments are obviously required to determine the long-term consequences of HPD on the colonic epithelium, taking into account that prolonged downregulation of the expression of genes associated with cell protection may be detrimental for colon mucosa health.

## Additional files

Additional file 1: Figure S1. Plot of the first and the second principal component (PC1 and PC2) of the PCA analysis of microarray data. a - Results of the PCA with all the samples (*n* = 16). #: Samples considered as outliers. b – Results of the PCA after removal of the two outliers (*n* = 14). Samples of rats fed a high-protein diet are in black and samples from rats fed a normal-protein diet are in red. (PDF 27 kb)
Additional file 2: Figure S2. Flow diagram of the transcriptome analysis in colonocytes of rats fed with a normal or a high-protein diet. a: for canonical pathways, Z-score statistics are calculated and indicate whether the canonical pathway is predicted to be activated (Z-score > 2) or inhibited (Z-score < 2). b: the *p*-value of overlap measures whether there is a statistical significant overlap between the set of genes differentially expressed between the two groups and the set of genes known to be associated with a given process or pathway. All the statistics were obtained with Ingenuity Pathways Software. (PDF 13 kb)
Additional file 3: Table S1. List of genes differentially expressed in colonocytes of rats fed a high-protein diet compared to rats fed a normal protein diet (q < 0.1).(.xls). (XLSX 122 kb)
Additional file 4: Figure S3. Validation of microarray data on differentially expressed genes of interest. Relative mRNA levels were measured by qPCR in colonocytes isolated from rats fed a normal-protein diet (NPD) or a high-protein diet (HPD). *Mt1a* (metallothionein 1A), *Sdc4* (syndecan 4), *Tfrc* (transferrin receptor), *Slc39a4* (solute carrier family 39 zinc transporter, member 4). Data presented are means +/- S.E.M. For each gene, mean values were compared with a *t* test. *: q < 0.05, ***: q < 0.001. (PDF 27 kb)
Additional file 5: Table S2. List of Ingenuity Pathway Analysis software biological categories and functions significantly enriched (*p* < 0.05) in the set of genes differentially expressed in colonocytes of rats fed a high-protein diet compared to rats fed a normal protein diet. (.xls). (XLSX 125 kb)
Additional file 6: Table S3. List of Ingenuity Pathway Analysis software canonical pathways significantly enriched (*p* < 0.05) in the set of genes differentially expressed in colonocytes of rats fed a high-protein diet compared to rats fed a normal protein diet. (.xls). (XLSX 67 kb)
Additional file 7: Figure S4.
*Integrin signaling* canonical pathway diagram. This pathway was significantly enriched in the set of genes regulated by the high-protein diet. The diagram was obtained from Ingenuity Pathway Analysis software and depicts genes implicated in this pathway and their interactions. Expression of genes colored in green were downregulated (q < 0.1) in colonocytes of rats fed a high-protein diet when compared to a normal-protein diet. (PDF 269 kb)
Additional file 8: Figure S5.
*Tight-junction signaling* canonical pathway diagram. This pathway was significantly enriched in the set of genes regulated by the high-protein diet. The diagram was obtained from Ingenuity Pathway Analysis software and depicts genes implicated in this pathway and their interactions. Expression of genes colored in green were downregulated (q < 0.1) in colonocytes of rats fed a high-protein diet when compared to a normal-protein diet. (PDF 465 kb)

## Abbreviations

DE: Differentially expressed
HPD: High-protein diet
NPD: Normal-protein diet
