# Supplementary figures and images for: Epithelial response to a high-protein diet in rat colon
Source: BMC Genomics. 2017 Jan 31;18:116. doi: 10.1186/s12864-017-3514-z (PMC5282643; doi:10.1186/s12864-017-3514-z)

Fig. S1

**A**

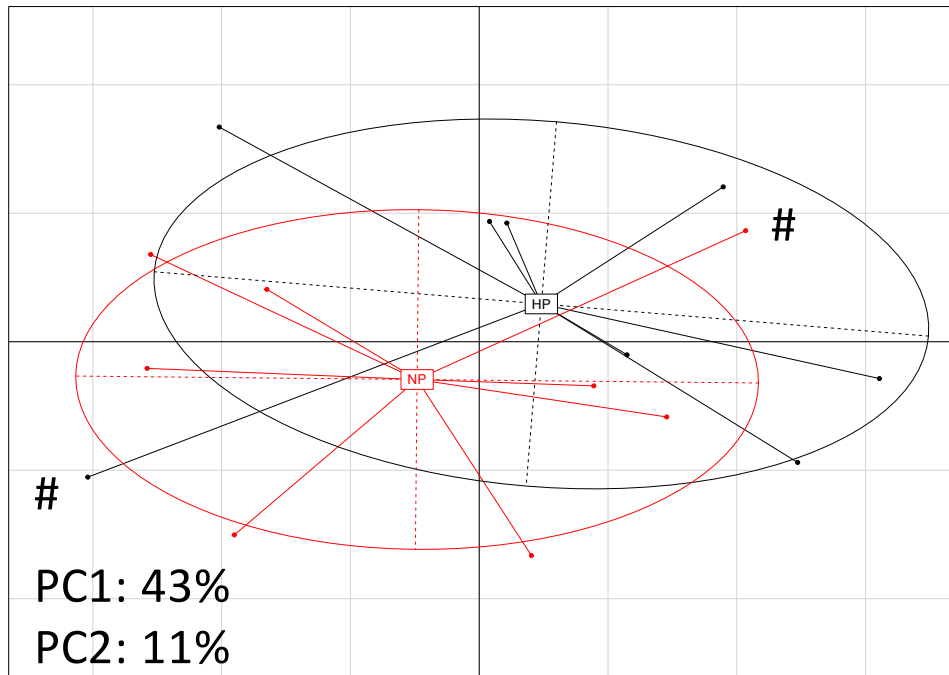

**B**

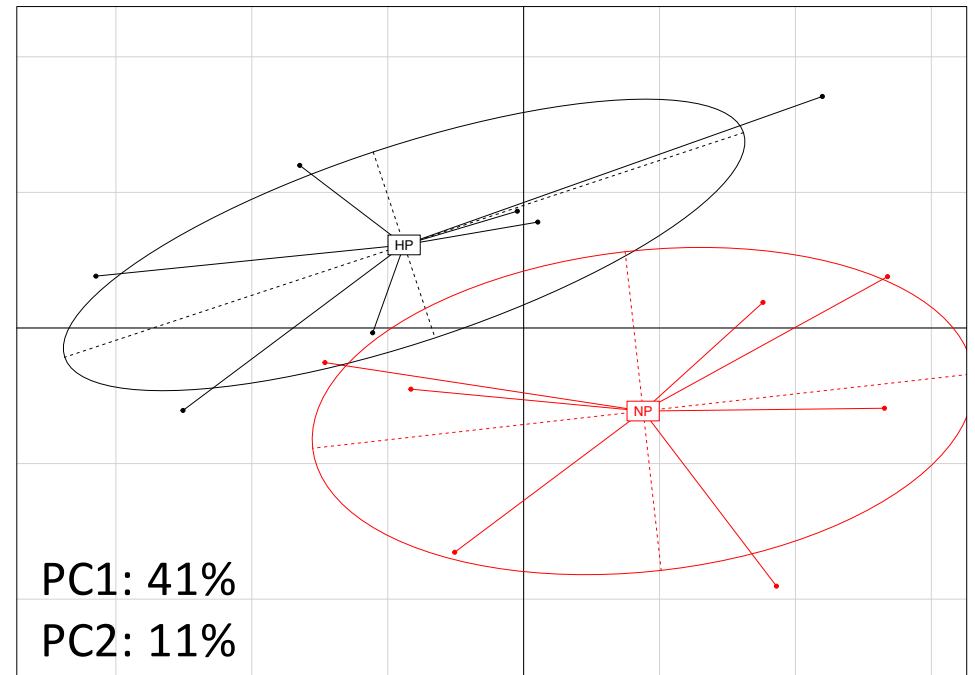

Supplement: Additional file 1: Figure S1. — Plot of the first and the second principal component (PC1 and PC2) of the PCA analysis of microarray data. a - Results of the PCA with all the samples (n = 16). #: Samples considered as outliers. b – Results of the PCA after removal of the two outliers (n = 14). Samples of rats fed a high-protein diet are in black and samples from rats fed a normal-protein diet are in red. (PDF 27 kb) [file 12864_2017_3514_MOESM1_ESM.pdf]

Fig. S2

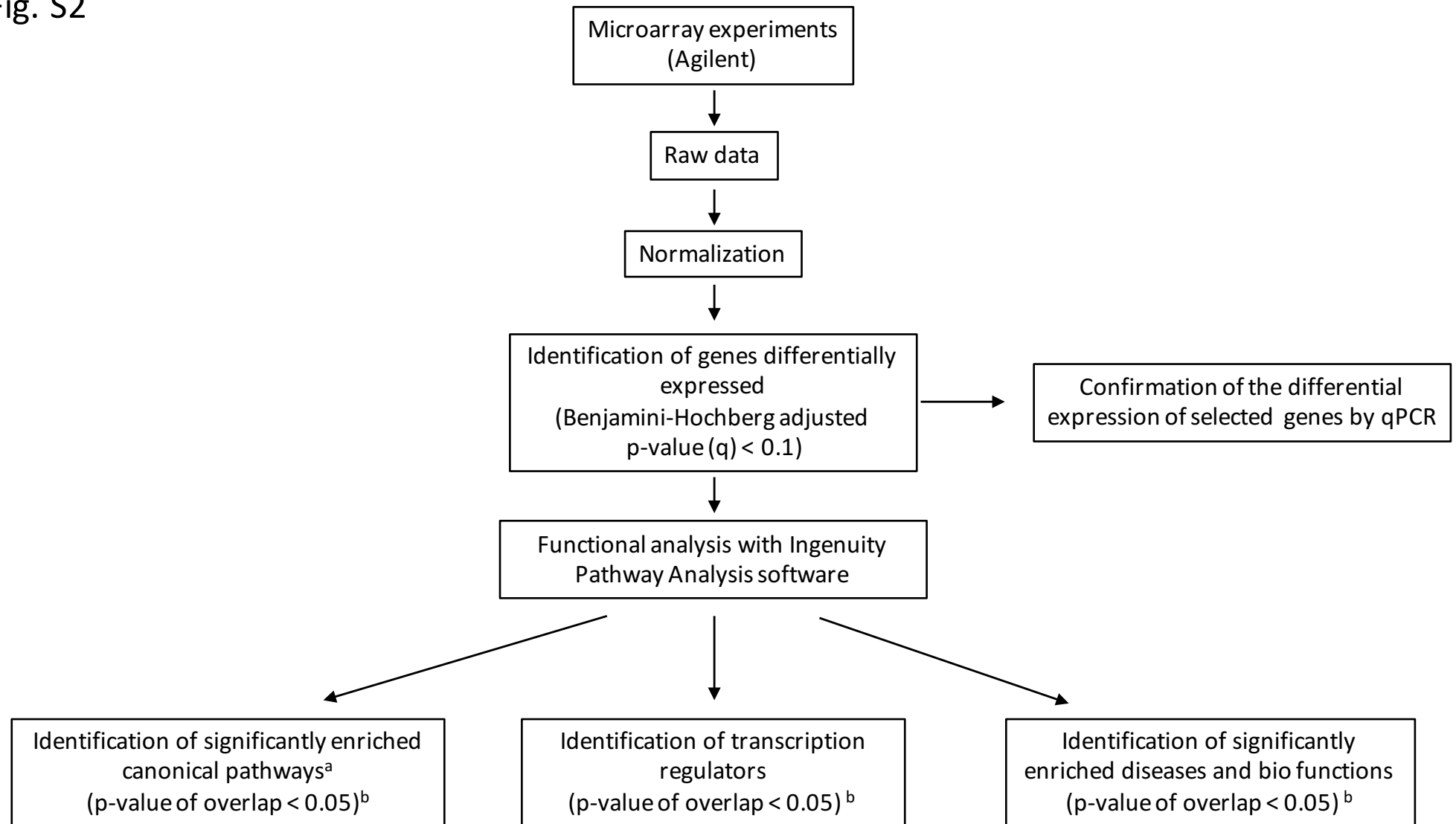

Supplement: Additional file 2: Figure S2. — Flow diagram of the transcriptome analysis in colonocytes of rats fed with a normal or a high-protein diet. a: for canonical pathways, Z-score statistics are calculated and indicate whether the canonical pathway is predicted to be activated (Z-score > 2) or inhibited (Z-score < 2). b: the p-value of overlap measures whether there is a statistical significant overlap between the set of genes differentially expressed between the two groups and the set of genes known to be associated with a given process or pathway. All the statistics were obtained with Ingenuity Pathways Software. (PDF 13 kb) [file 12864_2017_3514_MOESM2_ESM.pdf]

Fig. S3

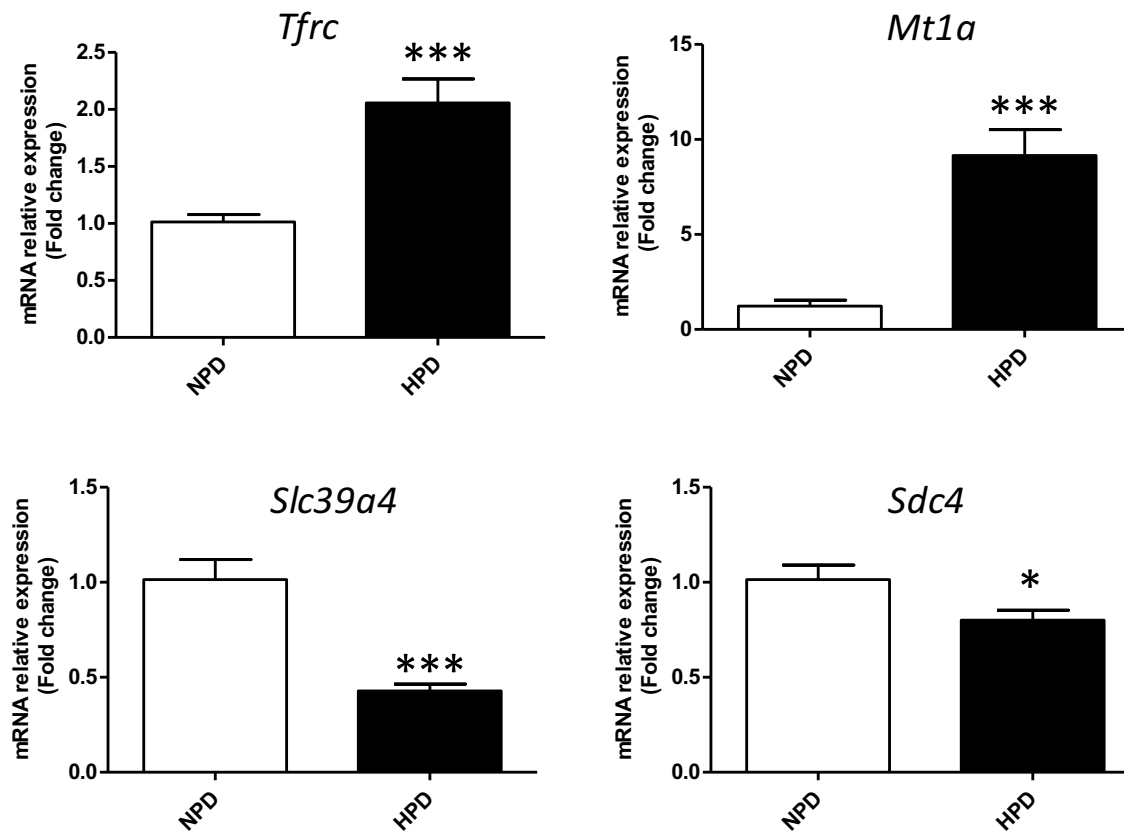

Supplement: Additional file 4: Figure S3. — Validation of microarray data on differentially expressed genes of interest. Relative mRNA levels were measured by qPCR in colonocytes isolated from rats fed a normal-protein diet (NPD) or a high-protein diet (HPD). Mt1a (metallothionein 1A), Sdc4 (syndecan 4), Tfrc (transferrin receptor), Slc39a4 (solute carrier family 39 zinc transporter, member 4). Data presented are means +/- S.E.M. For each gene, mean values were compared with a t test. *: q < 0.05, ***: q < 0.001. (PDF 27 kb) [file 12864_2017_3514_MOESM4_ESM.pdf]

Fig. S4

Integrin Signaling

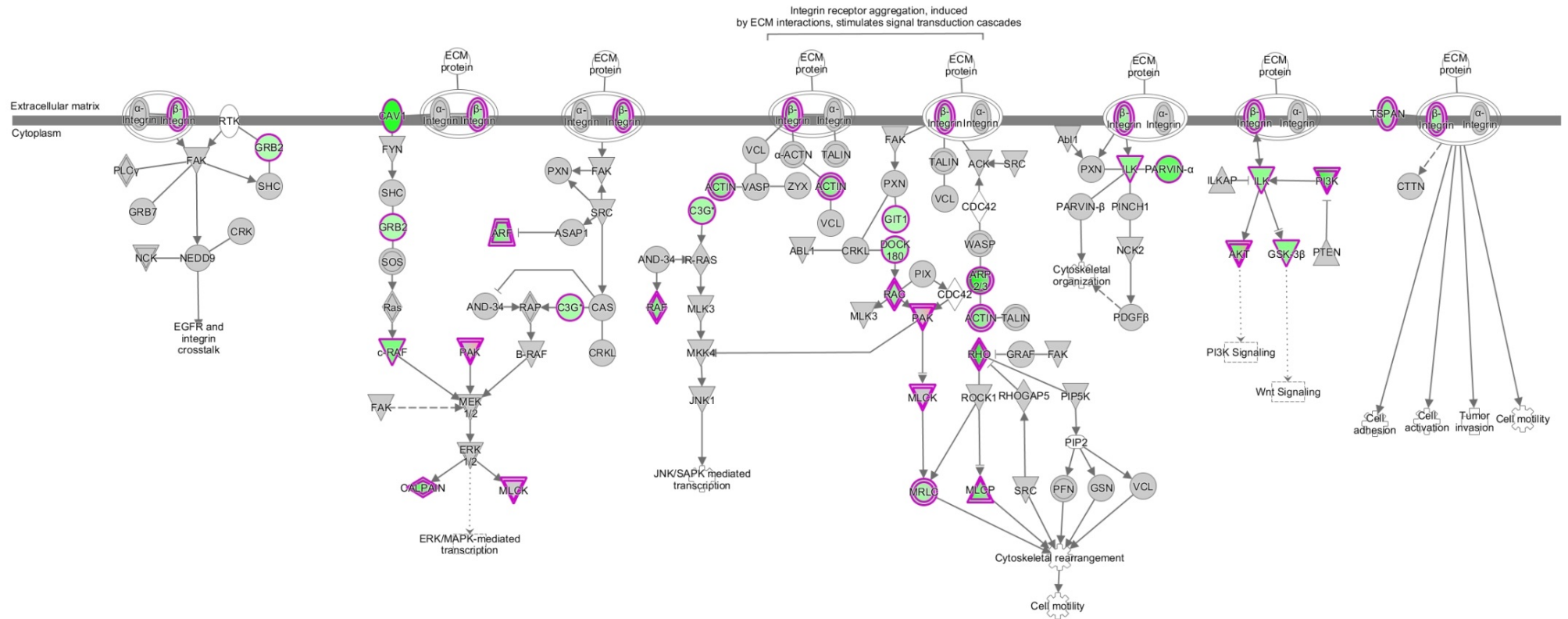

Supplement: Additional file 7: Figure S4. — Integrin signaling canonical pathway diagram. This pathway was significantly enriched in the set of genes regulated by the high-protein diet. The diagram was obtained from Ingenuity Pathway Analysis software and depicts genes implicated in this pathway and their interactions. Expression of genes colored in green were downregulated (q < 0.1) in colonocytes of rats fed a high-protein diet when compared to a normal-protein diet. (PDF 269 kb) [file 12864_2017_3514_MOESM7_ESM.pdf]

Fig. S5

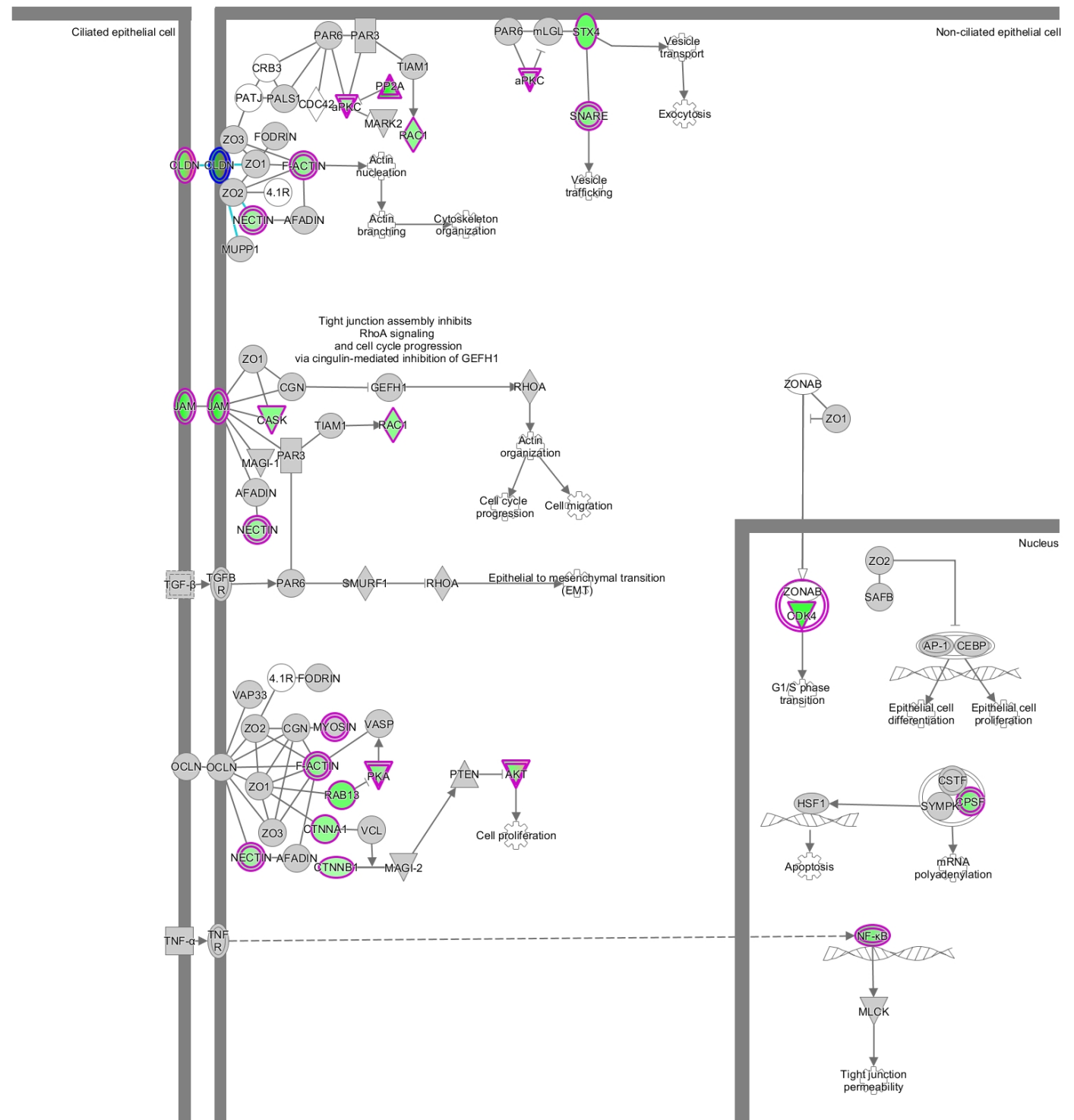

Supplement: Additional file 8: Figure S5. — Tight-junction signaling canonical pathway diagram. This pathway was significantly enriched in the set of genes regulated by the high-protein diet. The diagram was obtained from Ingenuity Pathway Analysis software and depicts genes implicated in this pathway and their interactions. Expression of genes colored in green were downregulated (q < 0.1) in colonocytes of rats fed a high-protein diet when compared to a normal-protein diet. (PDF 465 kb) [file 12864_2017_3514_MOESM8_ESM.pdf]
